# Baleen whale acoustic presence and behaviour at a Mid-Atlantic migratory habitat, the Azores Archipelago

**DOI:** 10.1038/s41598-020-61849-8

**Published:** 2020-03-16

**Authors:** Miriam Romagosa, Mark Baumgartner, Irma Cascão, Marc O. Lammers, Tiago A. Marques, Ricardo S. Santos, Mónica A. Silva

**Affiliations:** 1 Institute of Marine Research (IMAR) and Okeanos R & D Centre, University of the Azores, Horta, Portugal; 2 Biology Department, Woods Hole Oceanographic Institution, Woods Hole, MA USA; 3 NOAA’s Hawaiian Island Humpback Whale National Marine Sanctuary, Kihei, HI, USA and Oceanwide Science Institute, Honolulu, HI USA; 4 Centre for Research into Ecological and Environmental Modelling, University of St. Andrews, St. Andrews, UK and Centro de Estatística e Aplicações, Departamento de Biologia Animal, Faculdade de Ciências da Universidade de Lisboa, Lisboa, Portugal

**Keywords:** Behavioural ecology, Conservation biology

## Abstract

The identification of important areas during the annual life cycle of migratory animals, such as baleen whales, is vital for their conservation. In boreal springtime, fin and blue whales feed in the Azores on their way to northern latitudes while sei whales migrate through the archipelago with only occasional feeding. Little is known about their autumn or winter presence or their acoustic behaviour in temperate migratory habitats. This study used a 5-year acoustic data set collected by autonomous recorders in the Azores that were processed and analysed using an automated call detection and classification system. Fin and blue whales were acoustically present in the archipelago from autumn to spring with marked seasonal differences in the use of different call types. Diel patterns of calling activity were only found for fin whales with more calls during the day than night. Sei whales showed a bimodal distribution of acoustic presence in spring and autumn, corresponding to their expected migration patterns. Diel differences in sei whale calling varied with season and location. This work highlights the importance of the Azores as a migratory and wintering habitat for three species of baleen whales and provides novel information on their acoustic behaviour in a mid-Atlantic region.

## Introduction

Fin (*Balaenoptera physalus*), blue (*B. musculus*), and sei whales (*B. borealis*) were intensively hunted during the past century, drastically reducing their populations throughout their range. Despite the cessation of the majority of commercial whaling, their populations have not yet recovered and are still well below pre-whaling numbers^1^ Protection measures for these species are urgently needed, especially as human impacts in the marine environment continue to increase^2^. In the North Atlantic, fin, blue, and sei whales spend the summer at high-latitude feeding grounds and migrate to low latitudes during winter^3–7^. Knowledge about the location of these wintering areas, as well as of their migratory pathways and timing is still scarce. Identifying the full range of habitats used throughout the annual cycle and annual variation in habitat use is a key step in understanding the habitat requirements of these migratory species and critical for the development of effective conservation strategies.

**Figure 1:**
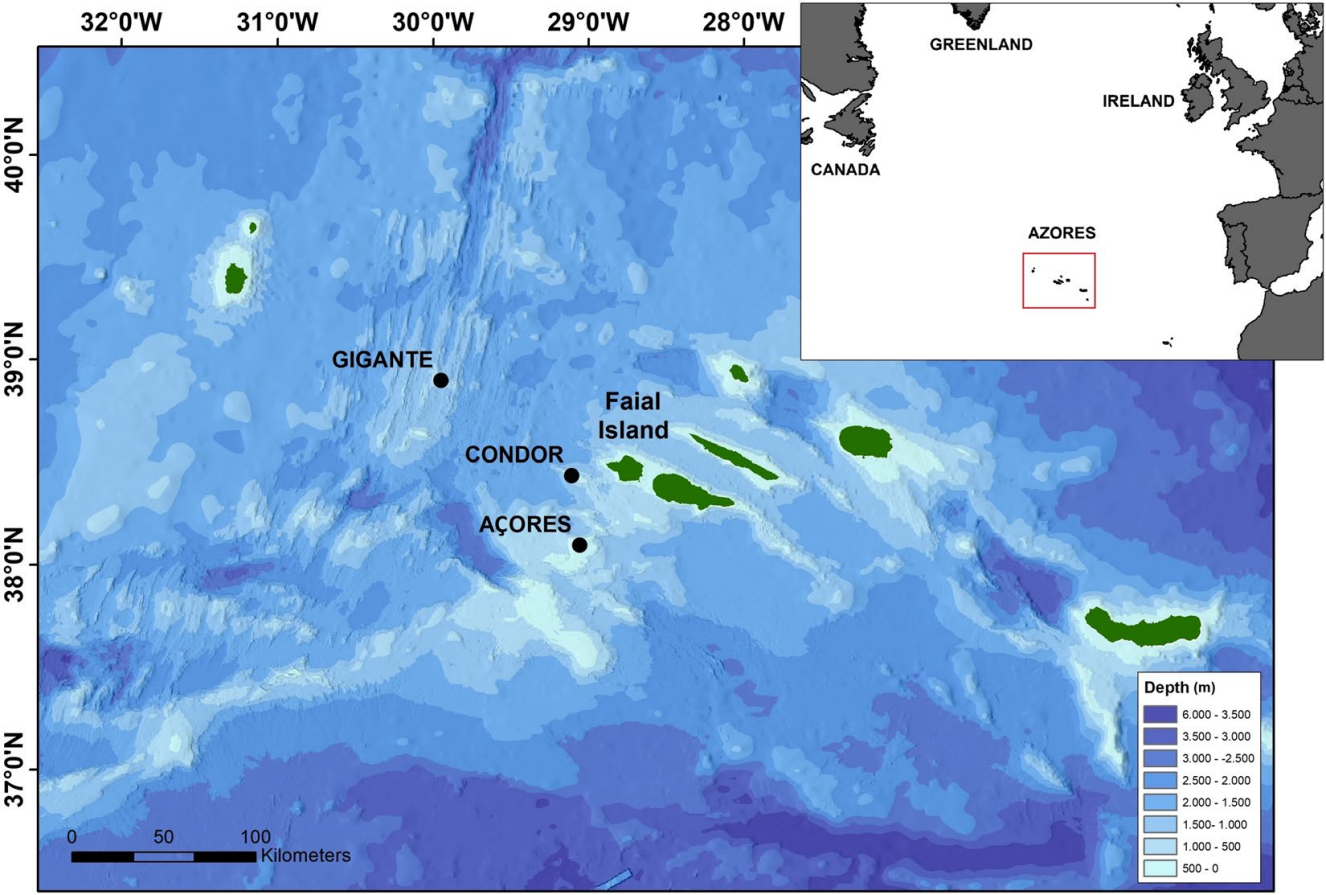
Location of the Azores (inset map) and of the hydrophone moorings at three seamounts: Açores, Condor and Gigante in relation to Faial Island. Figure produced with ArcGIS 10.1 (http://www.esri.com).

In the Azores Archipelago, located in the mid North Atlantic (Fig. 1), blue and fin whales stop to feed in spring while migrating to their high-latitude feeding areas^3^. Habitat suitability modelling indicates that the presence of both species in the area follows the spring bloom primary productivity^8^. As spring advances and favourable habitat conditions move progressively further north, fin and blue whales abandon the Azores, generally leaving the area in summer^8^ when chlorophyll a concentrations are at an annual minimum^9^. Sei whales seem to adopt a different strategy and transit through the archipelago quickly, feeding only occasionally, while on their way to the Labrador Sea^4,10^. Information on the presence of these species in the archipelago comes mostly from sighting data and satellite telemetry studies conducted during spring and summer months^3,11^. Occasional presence of fin, blue and sei whales in the Azores during autumn and winter is supported by a few sighting records^11^ but long-term, continuous observations are scarce mainly due to the offshore habits of these species and bad weather conditions during winter.

Passive acoustics is an excellent tool that enables continuous, long term monitoring of cetacean presence during all weather conditions^12^. Many studies have used blue, fin, and sei whale low-frequency calls to study seasonal presence^13,14^, long-term temporal trends^15^, migration patterns^16^, population structure^17,18^, behaviour^19,20^, distribution^21^, habitat use^22,23^ and abundance^24,25^. Each baleen whale species produces several call types, which can be used for monitoring their occurrence in different behaviours and seasons. Blue and fin whales produce calls in a regular pattern as part of a song, or sporadically as singular units or song fragments^26–29^. Songs are believed to act as reproductive displays because they are only produced by males^20,30^ and peak during the breeding season^26,31^, which happens in winter in the Northern Hemisphere^32^. However, blue whales have been reported to sing year-round in some locations with songs peaking during the summer on feeding grounds^33–35^ which suggests either a non-spatially and temporally restricted breeding strategy or a different use depending on context. Singular calls are often associated with social interactions or feeding behaviours^36,37^. Sei whales do not produce songs but they do produce very distinctive calls that occur in doublets or triplets that may act as contact calls between conspecifics^38^. The study of acoustic behaviour, such as seasonal segregation of different call types and their diel patterns, can provide clues to the functions of calls^36,39,40^, information about animal presence^33,41^ and an understanding of biases caused by non-vocalizing animals during specific periods of the day and year. The acoustic behaviour of fin and blue whales has been well studied in feeding areas of the north-eastern Pacific^39,42–44^, Antarctica^45,46^ and north-eastern Atlantic^16,47^ but few studies have focused in mid- and north-western Atlantic areas^28,48^ and even less in migratory temperate habitats. For sei whales, diel patterns in calling have only been investigated in the Gulf of Maine, a springtime feeding ground for this species^40^.

Here, we investigate the acoustic presence and behaviour of fin, blue and sei whales at a migratory habitat in the Mid-Atlantic, the Azores Achipelago. Using an acoustic dataset collected over 5 years, we describe the seasonal variability in the acoustic presence of these three species and the seasonal and diel patterns of their different calls.

## Results

### Vocalisations

**Figure 2:**
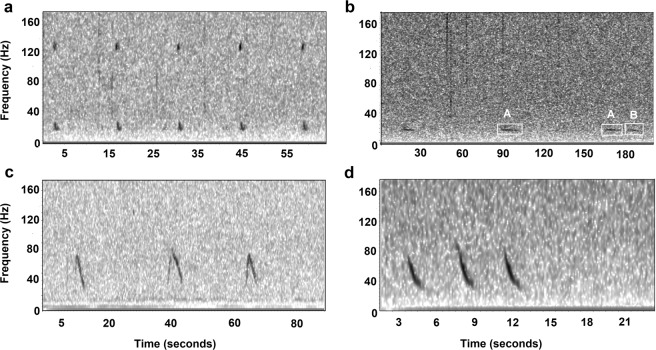
Example spectrograms with vocalizations of (**a**) fin whale double pulse calls, (**b**) blue whale A and AB calls (**c**), blue whale D calls and (**d**) sei whale downsweeps.

In total, 7009 hours of recordings were analysed from the deployments at Açores, Condor and Gigante seamounts. Fin whale 20 Hz pulses were the most commonly recorded baleen whale call. This pulse was found either alone (37% of fin whale calls and 34% of all species calls) or together with upsweeps between 130–135 Hz (63% of fin whale calls and 58% of all species calls) (double pulse call) (Fig. 2a). There was an order of magnitude fewer blue and sei whale calls detected as compared to fin whales. The most abundant blue whale call was the A call (81% of blue whale calls and 1.5% of all species calls) very rarely accompanied by a B part (AB calls) (5% of blue whale calls and 0.1% of all species calls) (Fig. 2b). Blue whale D calls were also detected sporadically in clusters (14% of blue whale calls and 0.2% of all species calls) (Fig. 2c). Only one type of sei whale call was detected, which was the characteristic downsweep call occurring in single, doublets or triplets (6.2% of all species calls) (Fig. 2d).

### Acoustic presence

**Figure 3:**
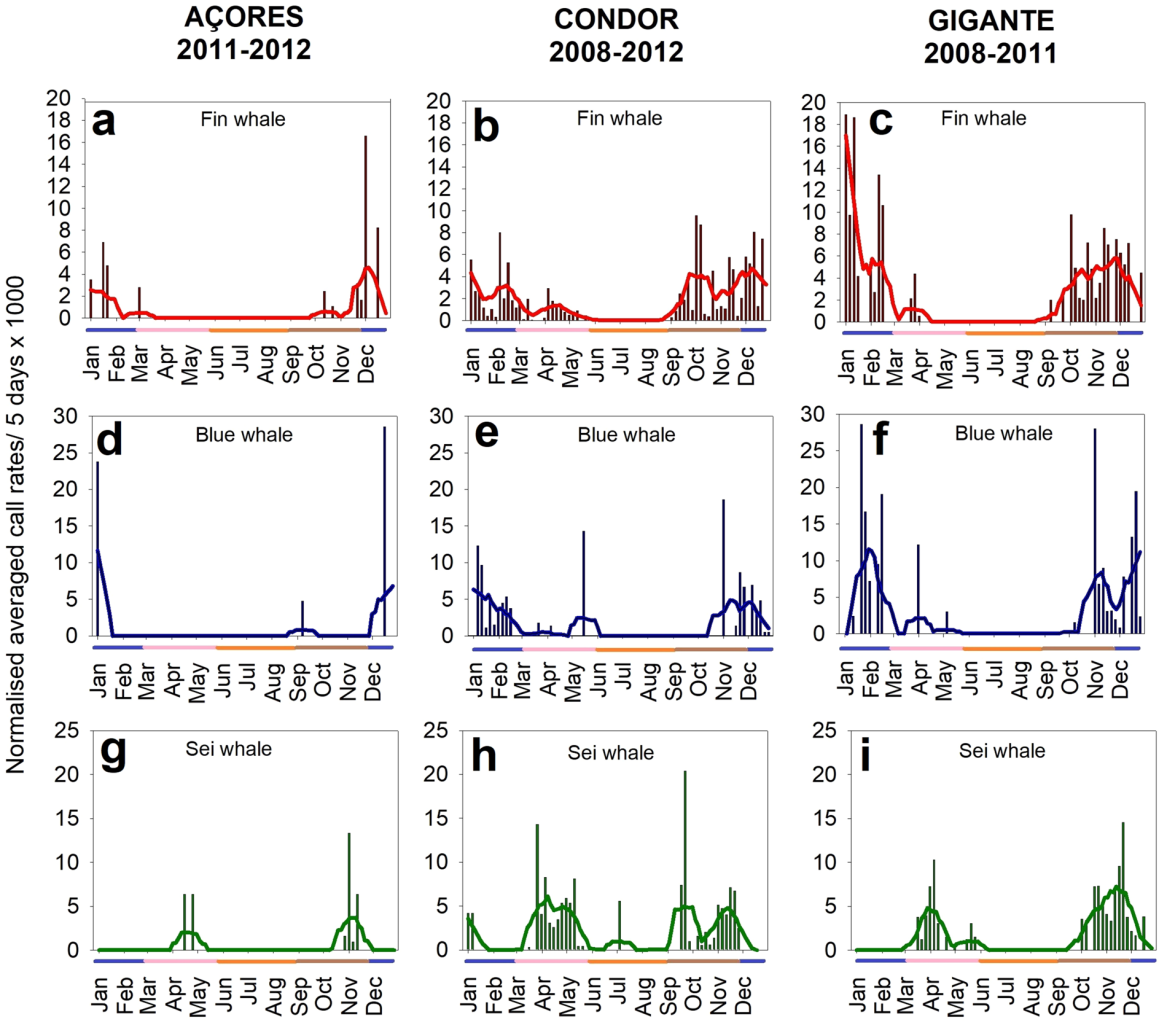
Seasonality of acoustic detections of fin (**a**–**c**), blue (**d**–**f**) and sei whales (**g**–**i**) in Açores, Condor and Gigante seamounts. Lines show smoothers (negative exponential) of data for a rapid interpretation of seasonal patterns. Coloured bar below the x-axis show seasons: blue for winter, pink for spring, orange for summer and brown for autumn.

Acoustic presence of fin, blue and sei whales showed a marked seasonality. A similar pattern was found across years and locations for both fin and blue whales, with increasing daily call rates in autumn, reaching a maximum in winter and decreasing again in spring with no detections in summer (Fig. 3a–c). Blue whale daily call rates (A-calls + AB-calls + D-calls) (Fig. 3d–f) increased slightly later (in winter) and decreased earlier (in spring) than fin whales. Sei whales showed a different pattern from that of fin and blue whales with number of calls peaking in spring and autumn in all locations (Fig. 3g–i). For a visual comparison of datasets, a complete time series for each location and species is provided as supplementary material (Supplementary Figs. S1–S3).

### Seasonal and diel patterns by call type

**Figure 4:**
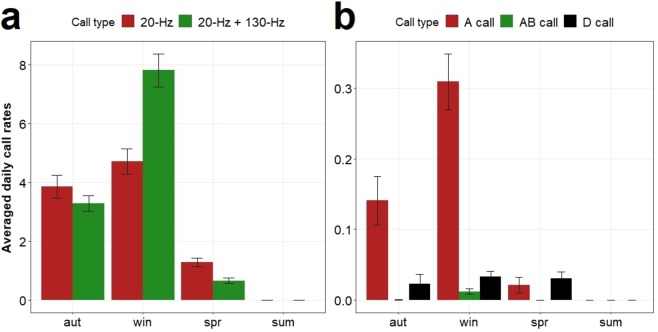
Averaged daily call rates per season at Condor and Gigante seamounts for fin (**a**) and blue whale (**b**) call types from 2008 until 2012. Error bars show standard errors. Seasons are defined as: aut – autumn, spr – spring, sum – summer and win – winter.

Fin and blue whale call types showed different seasonal patterns. The double pulse call was mostly produced in winter and was the most abundant fin whale call type at this time of the year. The 20-Hz pulse was detected mainly during winter and autumn and showed similar levels to the double pulse call in spring and autumn and much lower levels in winter (Fig. 4a). Blue whale A calls also showed a clear seasonality, with a peak in winter and decreasing in autumn and spring. AB calls were mostly found in winter and autumn. In contrast, D calls were detected at similar rates from autumn through spring (Fig. 4b).

Fin and blue whale call diel patterns did not show statistically significant differences between autumn and winter seasons (Supplementary Table S1) so they were grouped together for the analysis. Spring had a small number of days with detections (Condor: fin whale 20-Hz pulse = 48, fin whale double pulse call = 6, blue whale A call = 1 and blue whale D call = 2; Gigante: fin whale 20-Hz pulse = 12, fin whale double pulse call = 12, blue whale A call = 1 and blue whale D call = 2) so was not included in the analysis. Condor and Gigante data were analysed separately due to differences in the diel call patterns of these species.

**Figure 5:**
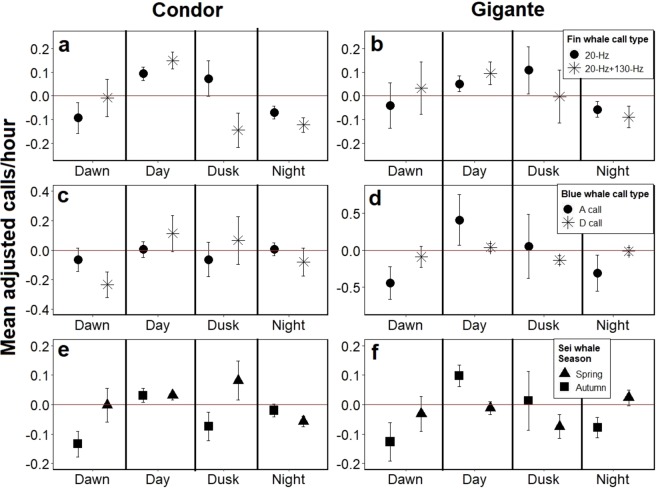
Mean adjusted number of calls and standard error by light regime of the fin whale 20-Hz pulse and the double pulse call for autumn and winter months in Condor (**a**) and Gigante seamounts (**b**), blue whale A and D calls for autumn and winter months in Condor (**c**) and Gigante seamounts (**d**) and sei whale downsweep call for spring and autumn in Condor (**e**) and Gigante (**f**).

Results from Kruskal-Wallis tests showed that call numbers are not the same for the four light regimes for both fin whale call types and seamounts (respectively for Condor and Gigante: 20-Hz: KW = 65.5, n = 912; KW = 40.4, n = 588 and double pulse call: KW = 66.4, n = 556; KW = 23.9, n = 336, all with probability P < 0.001). Dunn’s Multiple Comparison Test showed that day and night periods are significantly different from one another only in Condor, with more 20-Hz and double pulse calls emitted in daytime than in night-time (20-Hz pulse: Z = 3.4, n = 912, P < 0.01; double pulse call: Z = −4.1, n = 556, P < 0.001) (Fig. 5a). No differences were found between day and night periods for either call types in Gigante (20-Hz pulse: Z = 1.4, n = 588, P = 0.9; double pulse call: Z = 2.2, n = 336, P = 0.2) (Fig. 5b).

Blue whale A and D call numbers showed no differences between day and night-time for either Condor (Fig. 5c) or Gigante (Fig. 5d) (respectively for Condor and Gigante: A call: Z = −0.7, n = 128, P = 1; D call: Z = 0.4, n = 40, P = 1; A call: Z = −1.6, n = 24, P = 0.7; D call: Z = 0.4, n = 1, p-value = 88).

Sei whale diel call patterns showed differences between spring and autumn and between Condor and Gigante and so were analysed separately. In spring, sei whale call numbers are significantly higher during the day than during the night only in Condor (Z = 3.7, n = 192, P < 0.001) (Fig. 5e) while in autumn the same pattern occurs in Gigante (Z = −2.9, n = 172, P < 0.05) (Fig. 5f).

Dawn and dusk periods showed a great variance in call numbers with large standard errors of the mean so no clear pattern was found for these intermediate periods.

## Discussion

This is the first study showing that fin and blue whales are present in the Azores Archipelago during autumn and winter months. Whales were acoustically detected from September until May with no detections during the summer. A similar temporal pattern in the acoustic occurrence of fin and blue whales was found north and south of the Azores between latitudes of 50°N and 17°N, although detections were scarce south of 20°N^31,49^. Overall, this agrees with whaling and sighting records in the North Atlantic^7^ and suggests that fin and blue whales occupy a large offshore area in the mid North Atlantic during autumn and winter months^50^. The pattern of acoustic detections in spring but decreasing in summer agrees with visual studies documenting a peak in sighting rates from March to June, when whales are seen feeding, and few or no sightings during the summer^11^. However, the spring peak in sightings does not correspond to a peak in calling; call rates are much lower in spring than autumn or winter. Thus, fin and blue whales change their calling behaviour in spring, dramatically decreasing their call rates and/or switching their call types^13,37^, either because it is the end of the breeding season and/or because they spend more time foraging^3^. In the summer, the lack of blue whale calls can be explained by an absence of animals in the archipelago but fin whales may be present throughout the summer in small numbers depending on the year^11^ and remain undetected acoustically. Fin whales in the summer may be either silent or use other call types not identified in this study^13,37^. Sei whales show a different acoustic occurrence in the archipelago with two main peaks, one in spring and another one in autumn. This pattern agrees with the presumed migration of the species through the Azores, travelling north to the Labrador Sea during spring^4,10^ and south to possible breeding grounds in tropical waters during autumn^51^. We acknowledge that the calling seasonal patterns shown in this study are true only for the years sampled and that some variation may occur in other years.

Fin whale 20-Hz pulses reported in this study were either detected alone or with an upper frequency component, namely the double pulse call, which was mainly detected in autumn and winter months. The presence of these two call types in our recordings could be an artefact due to propagation loss. The double pulse call produced by distant whales may be detected as only a 20-Hz call because higher frequency sounds, like the 130-Hz component, suffer from higher attenuation with distance^52^. Besides, the 20-Hz pulses have 280 time more energy that the upper component^16^. If this was true, higher rates of double pulse calls detected in autumn and winter months could be the result of more fin whales being closer to the recorders during these seasons. However, this implies that fin whales may be further away from the recorders in spring, when the double pulse is more scarce, which is not supported by either visual^11^ nor satellite telemetry data^3^. Another hypothesis is that fin whales from the same population could be using two call types possibly linked to different behaviours. The double pulse call was mostly detected during the breeding season of fin whales which may represent a male reproductive display, as hypothesized for the 20-Hz pulse songs^26,30^. Unfortunately, our data from 2008–2011 has small duty cycles do not allow the identification of songs. Alternatively, two distinct acoustic populations could be producing these two call types. In the North Atlantic, this component, also referred as “135–140 Hz upsweep”, has been reported widely from east Greenland to the Alborán basin of the Mediterranean Sea^53–55^. To date, it has not been documented in the Northwest Atlantic. It is possible that some fin whales from the Northwest Atlantic frequent the Azores during the autumn and winter months. A recent study on stable isotopes identified the Iberian region as a winter feeding area for fin whales that visit the Azores in spring^56^ but no information exists on the origin of fin whales in the Azores during autumn and winter months.

Diel patterns of both fin whale call types indicate that more vocalisations are produced during the day than during the night. Although the same diel pattern occurs in both seamounts, it is only in Condor that differences between day and night periods are statistically significant. This contradicts other studies that report higher numbers of 20-Hz calls at night^16,23,26^ which have been associated with either a lower feeding activity during periods when krill is less aggregated^16^ or on the contrary, associated to feeding when herring fish densities are higher^23^. In the Azores, satellite tracking data showed enhanced swimming speeds for fin whales engaged in area-restricted search (ARS) behaviour (associated with feeding^57^) at night, with a clear peak at dawn and decreasing shortly after sunrise^3^. These authors suggest that fin whales feeding over deep waters may need to intensify their foraging effort at night to take advantage of the increased availability of diel vertically migrating prey in surface waters^3^. If we assume this to be true, then the lower numbers of both call types detected during the night coincide with a higher foraging activity of fin whales inferred from satellite telemetry. However, the fact that Gigante seamount differences between day and night call rates are not statistically different and the discrepancy between other studies^16,23^ may indicate that fin whale call diel patterns may vary depending on the animals’ behavioural state, feeding strategy or prey preferences. There is also the possibility of missing 130 Hz pulses due to the animals’ location. If this was the case, diel patterns of the double pulse call may not reflect the production of calls but the animal movements with respect to the recorders, as has been hypothesised by other authors in respect to diel patterns^58^. However, the fact that both call types show the same diel patterns makes this hypothesis seem unlikely.

Blue whale vocalizations, described for the first time in the Azores by this study, match the North Atlantic call type, recorded throughout the North Atlantic including the Mid-Atlantic Ridge^49^ and the Northeast^59^ and Northwest Atlantic^27,28,60^. In this study, AB calls were rarely detected compared to A calls (5.9% of calls). This could be a consequence of a) B calls with lower source levels not being detected by the EARs lower sensitivity below 18 Hz and/or b) missing calls caused by small duty cycles (2008–2011) or c) a true low number of AB calls. While a similar pattern had been reported in the Mid-Atlantic Ridge^49^ and the Gulf of Saint Lawrence^27^ (with a higher percentages of AB calls than in the Azores: 29% and 23% respectively), the opposite had been found for a large offshore area of the Northwest Atlantic where AB calls were the most recorded call (65.7%) compared to A calls (33.7%)^28^. These differences do not match photo-identification data that suggest the existence two largely discrete blue whale populations in the North Atlantic (Northeast Atlantic and Northwest Atlantic)^61^. This could be due to: a) different uses of these specific call types are not linked to population identity or b) differences between recorders sensibilities affecting the detectability of B parts. Temporal differences in the production of the three call types, A, AB and D, indicate they may be used in different contexts. A and AB calls were mainly present in autumn and winter months^62^, which agrees with previous studies showing that regularly repeated A or A-B calls forming songs were produced during the hypothesized blue whale breeding season^41,49^. In this study though, we cannot differentiate if calls were forming songs or not due the small duty cycles used from 2008 to 2011. D calls were detected in all seasons, except summer, which may relate to the potential multifunctionality of this call. The use of D calls have been described in varying behavioural contexts that include from foraging^20,39^ to social interactions^36,44^ and even in competitive behaviour linked to reproduction^63^.

Diel patterns of blue whale calling activity did not show any significant differences between day or night periods for either A or D calls. Many studies conducted in the North Pacific Ocean have reported a higher number of blue whale A, B or A-B songs during the night, possibly coinciding with lower feeding activity^20,36,42,64^. The lack of a clear diel pattern in our study may be the result of either the inability to distinguish song fragments from songs due to our duty-cycled data or a true absence of a diel pattern. Blue whale D calls also showed little variation between light regimes. Other studies reported different diel patterns for this call, with more D calls during the day in the North Pacific^36^ and during the night in the Northwest Atlantic^23^. D calls have been linked to periods of higher feeding activity but are more likely to be contact calls than foraging calls^20,36,44^. New data suggest that D calls could even be produced in reproductive contexts of male competition^63^.

Sei whale downsweep calls found in this study had been previously described in the Azores^65^ and showed strong similarities with the ones described in the Northwest Atlantic^38^. The lack of regional call differences between these two areas agree with satellite and genetic studies indicating that western and central North Atlantic sei whales are part of the same population^4,66^. Sei whales found off the Gulf of Maine vocalize more during the day than at night^40^. Sei whales feed on surface aggregations of highly migrant zooplankton (mainly copepods) during the night, and higher calling activity during the day may serve a social function, maybe to advertise high density prey patches to conspecifics^40^. Detection of sei whale downsweeps in the Azores exhibited the same diel pattern as documented in the Gulf of Maine only in spring at Condor seamount and in autumn at Gigante seamount. This is an interesting result, because behavioural observations, satellite telemetry and stable isotope analysis all indicate that sei whales forage only sporadically in the Azores^4,11^. Either sei whales feed more often than detected by current observations in those seamounts and seasons and/or downsweeps are not strictly associated with feeding activity and may be also used as contact calls during migration and their diel patterns are affected by unknown variables.

This work emphasizes the importance of the Azores for three species of baleen whales. First, it places the archipelago as part of a large wintering area for fin and blue whales in the mid North Atlantic Ocean. Second, it confirms the relevance of the Azores as a migratory area for sei whales in spring and autumn.

Given the seasonality of these species in the archipelago, a spatial management approach that takes into account a temporal dimension should be considered as the most appropriate conservation strategy. Impacts known to cause disturbance to these species in the short and long-term should be regulated in space and time by integrating near-real time biological information such as habitat use. Noise produced by intense shipping and oil and gas exploration overlaps with baleen whale vocalisations and is known to cause behavioural responses to fin and blue whales^67,68^ which, in the long term, could displace them or affect their survival. More research is needed in autumn and winter months to identify the spatial distribution of fin and blue whales in the Azores as well as the environmental drivers of their presence.

## Methods

### Survey area and acoustic recordings

Bottom-mounted Ecological Acoustic Recorders (EARs)^69^ were moored at the base of three seamounts in the Azores: Açores, Condor and Gigante (Fig. 1) at approximately 190 m depth. Açores seamount, located 40 km south-west from Faial Island, is characterized by a large flat summit with shallow surrounding depths (190–500 m) due to the proximity of other shallow banks to the south. Condor seamount, located 17 km to the WSW of Faial Island, is a two peak shallow-intermediate seamount with a nearly flat summit of 11.6 km^2^, steeper slopes and deeper surrounding depths ranging from 700 to 1500 m^70^. Gigante seamount is 98 km to the WNW of Faial Island and 6 km east of the Mid-Atlantic Ridge. It is a shallow seamount with a small summit of 0.7 km^2^ reaching 161 m depth, steep slopes and surrounding depths similar to Condor seamount.

**Figure 6:**
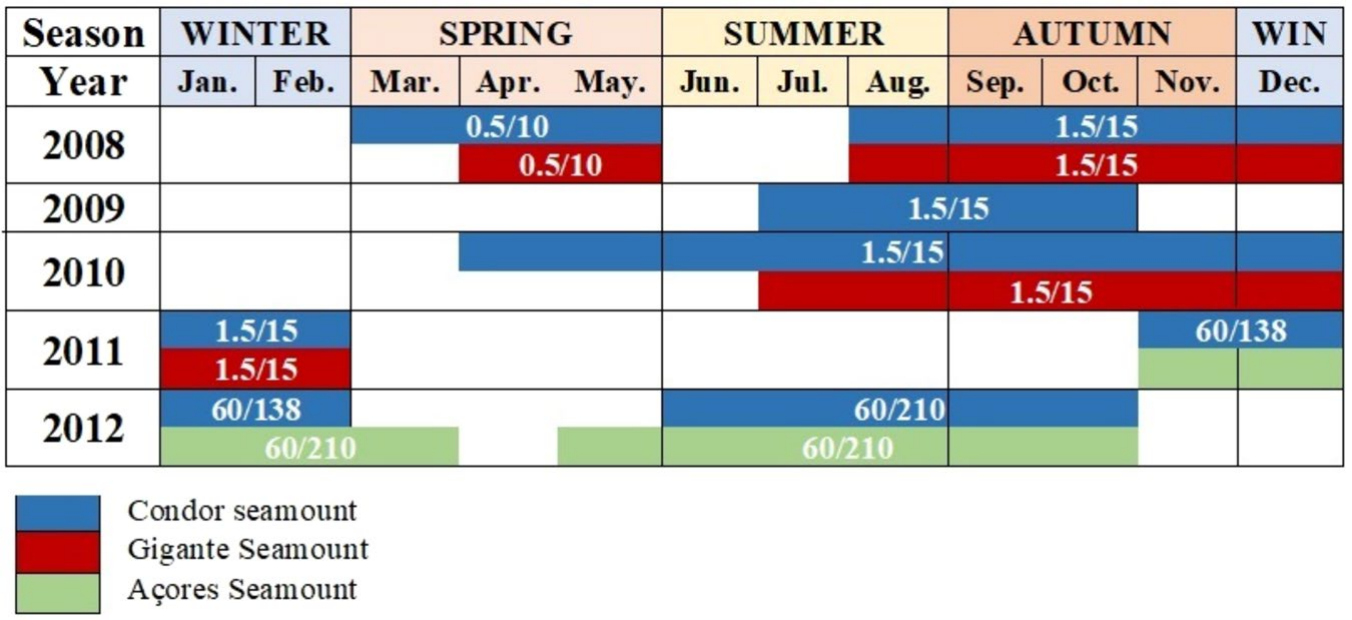
Deployment times and duty cycles for each season, month and seamount. Duty cycles for each deployment are in white numbers and indicate recording periods (min on/min off).

The EAR consists of a Sensor Technology SQ26-01 hydrophone with a response sensitivity of −193.14/−194.17 dB re 1 V/μPa (varying between deployments) for Açores and Condor and −193.64/−193.14 dB for Gigante and a flat frequency response (±1.5 dB) from 18 Hz to 28 kHz. Deployments were set to different duty cycles and sample rates due to multispecies studies and constraints of battery life and data storage capacities. Sampling rates of recordings from Gigante (all deployment) and Condor (March 2008 - February 2011) were of 50 kHz and from Açores (all deployment) and Condor (November 2011 - October 2012) were of 2 kHz. Longer duty cycles (60 min on/138 min off and 60 min on/210 min off) cycled over time so not the same time was recorded every day. Gaps of acoustic recordings found in the time series were caused by maintenance duties and equipment failure. However, all four seasons are well represented in three of the five years sampled (2008, 2010 and 2012) (Fig. 6).

### Automatic detection of calls

Given the extensive acoustic dataset of this study, a Low Frequency Detection and Classification System (LFDCS)^71^ was used to automatically detect and classify calls from fin, and sei whales. A precursor step was the development of a reference call library that contained known calls from fin and sei whales, previously identified and manually extracted from the dataset. Two types of calls from fin whales were included: the 20-Hz pulse, a 1 second downsweep centred at 20 Hz^26^, and the 130-Hz upsweep, a higher frequency note from 130 Hz to 140 Hz^16,53^. Only one type of call was included for sei whales, the downsweep call, which sweeps from 83 Hz to 34 Hz lasting about 1.4 s^38^. To better visualize how well different call types in the library were separated, scatterplots of attributes of each call type were plotted against one another (Supplementary Fig. S4). EAR recordings were downsampled to a sample rate of 2000 Hz to obtain standardized data covering the frequency range of interest and then processed by the LFDCS. Spectrograms were smoothed using a Gaussian kernel and tonal and broadband noise removed. The resulting filtered spectrograms were then used to find candidate tonal calls using an amplitude threshold. When a candidate call was found, the LFDCS estimated a pitch-track, which characterizes the frequency and amplitude variation of the call over time. For each pitch-track, seven amplitude-weighted attributes were compared to those of each call type in the reference call library, using a quadratic discriminant function analysis (QDFA). The “quality” of a match between the pitch-track and a call type in the call library was assessed with the Mahalanobis distance^72^, which is the distance between the new call and the QDFA-classified call type in the reference library. A previous preliminary manual analysis of the entire dataset, which identified files with and without detections of each call, allowed us to improve the results from the LFDCS by removing false positives detections. Blue whale calls were identified and counted manually due to their low abundance by comparing them with available literature^28,49^. Calls were differentiated from tonal noise because they decreased in frequency and did not occupy the full file. Even in smaller duty cycled recordings with files that lasted only 30 seconds, we could distinguish separate A calls, which lasted for 17 seconds.

To assess the performance of the LFDCS, results from one month of recordings were manually analysed for fin and sei whale calls, by logging calls missed by the detector and false positive detections. Months selected for each species were representative in terms of background noise during the rest of the months and years. Potential bias caused by varying background noise levels across months was reduced by spectrogram conditioning which eliminated tonal and broadband noise. Variability in call rates across seasons was reduced by removing false positives. Classifier performance was evaluated using a receiver operating characteristic (ROC) curve as a function of the Mahalanobis distance value. The percentage of false positives (false calls selected by the detector divided by the total number of detections) was plotted against the percentage of true positives (true calls detected by the detectors divided by the total known true calls in the dataset) for each Mahalanobis distance (Supplementary Fig. S4). The chosen Mahalanobis distance was the one that gave the best compromise between false and true positives and false negatives or missed calls (missed true calls by the detector divided by the total number of known true calls in the dataset). A maximum Mahalanobis distance of 5 was used for detecting fin whale 20-Hz (false positives: 0.9%; true positives: 80%; missed calls: 20%) and 130-Hz upsweeps (false positives: 0.7%; true positives: 85%; missed calls: 34%) and 4 for sei whale downsweeps (false positives: 2.7%; true positives: 66%, missed calls: 34%).

### Statistical analysis

Assuming that calling behaviour is equally distributed throughout an hour, a correction was applied to call rates to account for the different duty cycles used in this study. Thus, daily call rates were calculated as the total number of detected calls per day divided by the length of recording time during that day (daily call rates/hour). To account for the inter-annual variability we normalised data by dividing each daily call rate by the sum of calls of the corresponding year. Seasonality in the acoustic presence of each species was investigated by averaging the normalized daily call rates over a 5-day period across all years for each whale species (grouping each species call types) and seamount. Seasonality of each call type was examined by averaging daily call rates per season across the three locations. Months were assigned to meteorological seasons reckoned by temperature. In this study, this assignment worked well with the acoustic baleen whale presence in the Azores and the different call usage. Seasons were defined as follows: Spring: March–May, Summer: June–August, Autumn: September–November and Winter: December–February.

Diel patterns for each call type and species were investigated by sorting detections into four light regimes (dawn, day, dusk and night) based on the altitude of the sun, which was obtained from the United States Naval Observatory Astronomical Applications Department website (http://aa.usno.navy.mil). Dawn hours start when the sun is 12° below the horizon and finish at sunrise when light hours start. Dusk corresponds to the period after sunset until the sun is 12° below the horizon. Dark hours are between dusk and dawn. Only days with detections and data with duty cycles covering all hours (Condor and Gigante deployments 2008–2011) were used. Because the duration of light regimes differ and vary over the course of one year, daily number of calls in each light period were divided by the duration of the corresponding time period providing normalized detection rates (detections/hour) for each light regime. Given the variation in the number of calls among days, the resulting normalized detection rates for each light regime and for each day were adjusted by subtracting the mean number of calls during that day^42,64^. To investigate if the number of detections per hour differ between light regimes, we conducted the non-parametric test Kruskal-Wallis followed by a Dunn’s multiple comparison test with a Bonferroni adjustment method^73^. This test was chosen because data did not follow a normal distribution. Both tests assume independence of observations so data were transformed prior to testing to correct for serial correlation. First, an autocorrelation function was used to check for data autocorrelation and then an auto regressive integrated moving average (ARIMA) model was fitted only to data showing autocorrelation. In each case, the AR order from the ARIMA model was adjusted until getting rid of the autocorrelation. The resulting residuals from the model were used in the Kruskall-Wallis and Dunn’s multiple comparison test. This analysis was done with nlme package in R software version 5.4^74^.

## Supplementary information

Supplementaryinformation

## Data Availability

The datasets generated during and/or analysed during the current study are available from IMAR – Institute of Marine research by contacting the corresponding author on reasonable request.
