# Author Correction: Baleen whale acoustic presence and behaviour at a Mid-Atlantic migratory habitat, the Azores Archipelago

**DOI:** 10.1038/s41598-020-63117-1

**Published:** 2020-04-03

**Authors:** Miriam Romagosa, Mark Baumgartner, Irma Cascão, Marc O. Lammers, Tiago A. Marques, Ricardo S. Santos, Mónica A. Silva

**Affiliations:** 1 Institute of Marine Research (IMAR) and Okeanos R & D Centre, University of the Azores, Horta, Portugal; 2 Biology Department, Woods Hole Oceanographic Institution, Woods Hole, MA USA; 3 NOAA’s Hawaiian Island Humpback Whale National Marine Sanctuary, Kihei, HI, USA and Oceanwide Science Institute, Honolulu, HI USA; 4 Centre for Research into Ecological and Environmental Modelling, University of St. Andrews, St. Andrews, UK and Centro de Estatística e Aplicações, Departamento de Biologia Animal, Faculdade de Ciências da Universidade de Lisboa, Lisboa, Portugal

Correction to: *Scientific Reports* 10.1038/s41598-020-61849-8, published online 16 March 2020

This Article contains typographical errors in the Acknowledgements section.

“through research projects TRACE (PTDC/MAR/74071/2006), MAPCET (M2.1.2/F/012/2011) and AWARENESS (PTDC/BIA-BMA/30514/201), co-funded by FEDER, COMPETE, QREN, POPH, ERDF, ESF, the Lisbon Regional Operational Programme, and the Portuguese Ministry for Science and Education. Funding for publication fees was provided by Project AWARENESS (PTDC/BIA-BMA/30514/201)”

should read:

“through research projects TRACE (PTDC/MAR/74071/2006), MAPCET (M2.1.2/F/012/2011) and AWARENESS (PTDC/BIA-BMA/30514/2017), co-funded by FEDER, COMPETE, QREN, POPH, ERDF, ESF, the Lisbon Regional Operational Programme, and the Portuguese Ministry for Science and Education. Funding for publication fees was provided by Project AWARENESS (PTDC/BIA-BMA/30514/2017)”

