# Supplementary material for: Baleen whale acoustic presence and behaviour at a Mid-Atlantic migratory habitat, the Azores Archipelago
Source: Sci Rep. 2020 Mar 16;10:4766. doi: 10.1038/s41598-020-61849-8 (PMC7075977; doi:10.1038/s41598-020-61849-8)
Supplement: Supplementary file 1 — Supplementaryinformation [file 41598_2020_61849_MOESM1_ESM.docx]

**Baleen whale acoustic presence and behaviour at a Mid-Atlantic migratory habitat, the Azores Archipelago**

Miriam Romagosa*^1^, Mark Baumgartner^2^, Irma Cascão^1^, Marc O. Lammers^3^, Tiago A. Marques^4^_,_ Ricardo S. Santos^1^, Mónica A. Silva^1,2^ Marine and Environmental Sciences Centre (MAR E) Horta, Portugal

2. Biology Department, Woods Hole Oceanographic Institution, Woods Hole, MA, USA

3. NOAA's Hawaiian Island Humpback Whale National Marine Sanctuary, Kihei, HI, USA and Oceanwide Science Institute, Honolulu, HI, USA

4. Centre for Research into Ecological and Environmental Modelling,University of St. Andrews, St. Andrews, UK and Centro de Estatística e Aplicações, Departamento de Biologia Animal, Faculdade de Ciências da Universidade de Lisboa, Lisboa, Portugal

**SUPPLEMENTARY FIGURES**

**
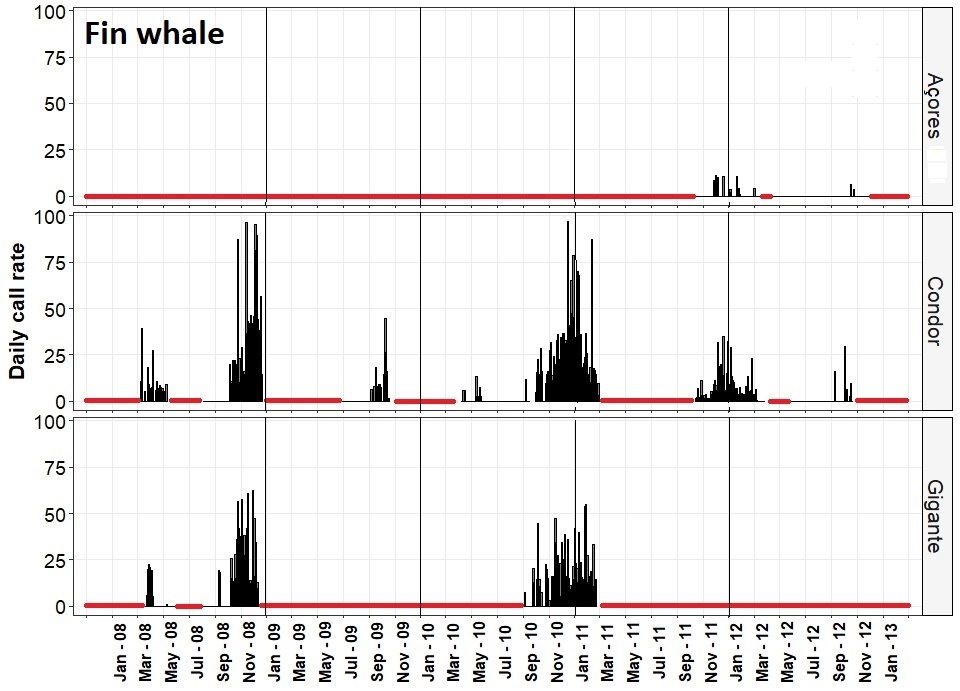
**

Figure S1. Fin whale daily call rates in Açores, Condor and Gigante seamounts from 2008 until 2012. Red line shows periods with no data.


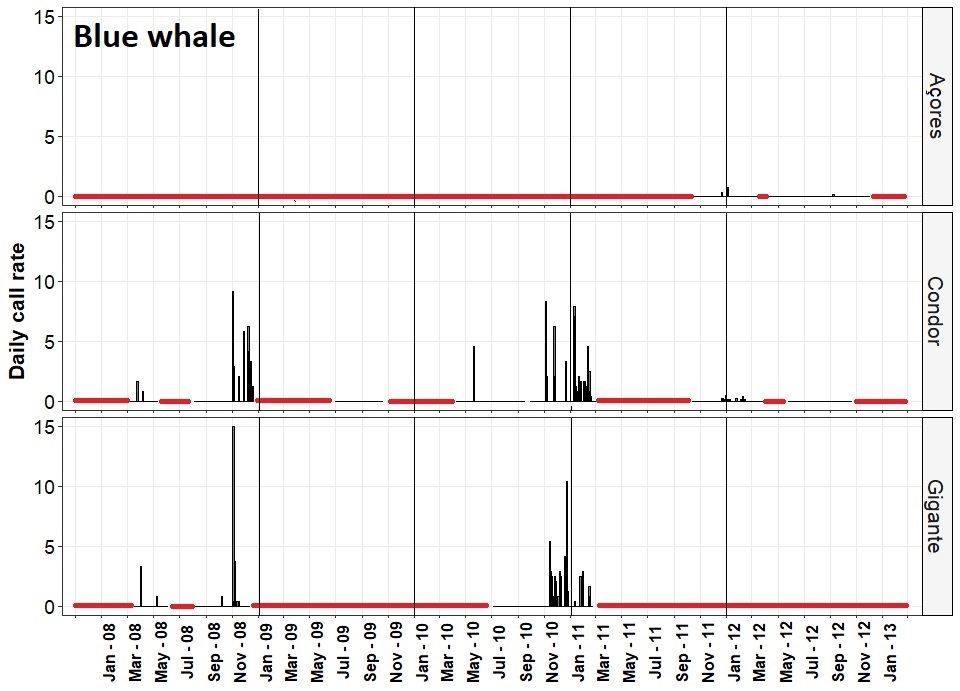


Figure S2. Blue whale daily call rates in Açores, Condor and Gigante seamounts from 2008 until 2012. Red line shows periods with no data.


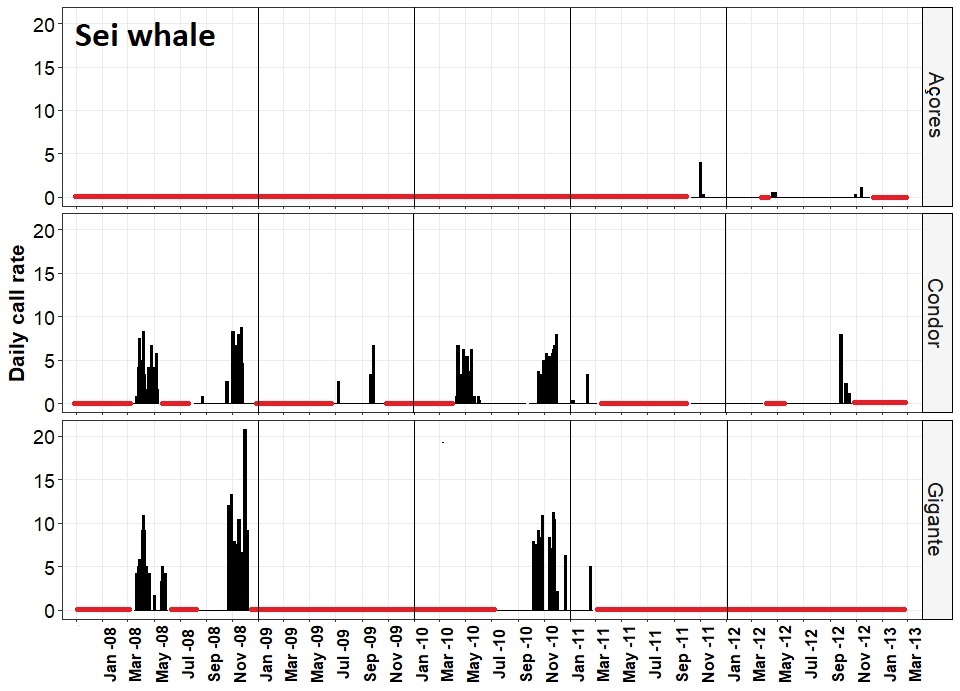


Figure S3. Sei whale daily call rates in Açores, Condor and Gigante seamounts from 2008 until 2012. Red line shows periods with no data.


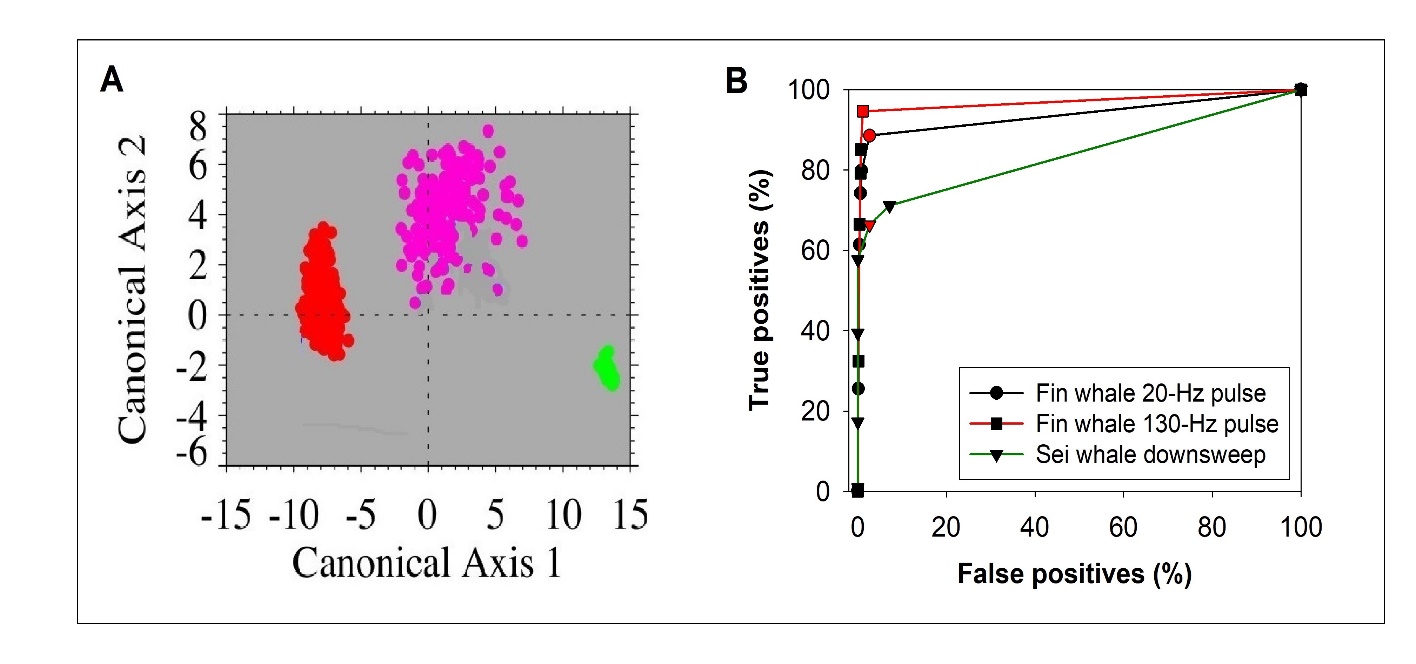


Fig. S4. (A) Scatterplots for fin whale 20-Hz pulse (red), 130-Hz upsweep (green) and sei whale downsweep (pink) resulting from a canonical discriminant function analysis. For better visualization, scatterplots of attributes of each call type were plotted against one another by reducing the 7 attribute dimensions down to 2 through a canonical discriminant functional analysis (CDFA), (B) ROC curves showing the performance of the LFDCS for varying Mahalanobis distance (0 – >5) for calls of fin and sei whales. Red dots show the chosen Mahalanobis distance for this study.

**SUPPLEMENTARY TABLE**

Table S1. Results form Kruskal-Wallis tests comparing diel patterns of autumn and winter for each seamount and species call.

| **KW test for diel patterns of autumn and winter months** | **CONDOR** | **GIGANTE** |
| --- | --- | --- |
| **Fin 20-Hz call** | Naut.=548, Nwin.=364  Chi square= 0.05, df=1, p-value=0.82 | Naut.=472, Nwin.=452  Chi square = 0.3, df=1, p-value=0.1 |
| **Fin whale 20-Hz + 135-Hz call** | Naut.=280, Nwin.=276  Chi square = 0.08, df=1 p-value=0.8 | Naut.=160, Nwin.=176  Chi square = 0.05, df=1p-value=0.82 |
| **Blue whale A call** | Naut=40, Nwin=128  Chi square = 1.5, df=1, p-value=0.2 | Naut=40, Nwin=72  Chi square = 0.7, df=1, p-value=0.4 |
| **Blue whale D call** | Naut=16 Nwin=24  Chi square = 4.6, df=1, p-value=0.05 | Naut=28 Nwin=60  Chi square = 1.5, df=1, p-value=0.21 |
